# Characteristics of inhomogeneous lower extremity growth and development in early childhood: a cross-sectional study

**DOI:** 10.1186/s12887-021-02998-1

**Published:** 2021-12-06

**Authors:** Sudarat Apibantaweesakul, Shiho Omura, Weihuang Qi, Hiroto Shiotani, Pavlos E. Evangelidis, Natsuki Sado, Fumiko Tanaka, Yasuo Kawakami

**Affiliations:** 1 Graduate School of Sport Sciences, Waseda University, Saitama, 359-1192 Japan; 2 Department of Sports Science and Sports Development, Faculty of Allied Health Sciences, Thammasat University, Pathum Thani, 12121 Thailand; 3 Waseda Institute for Sport Sciences, Waseda University, Saitama, 359-1192 Japan; 4 Faculty of Sport Sciences, Waseda University, 2-579-15 Mikajima, Tokorozawa, Saitama, 359-1192 Japan; 5 Faculty of Health and Sport Sciences, University of Tsukuba, Ibaraki, 305-8574 Japan; 6 Human Performance Laboratory, Comprehensive Research Organization, Waseda University, Saitama, 359-1192 Japan

**Keywords:** Bone growth, Muscle thickness, Adiposity, Isometric torque, Ultrasound

## Abstract

**Background:**

Early childhood is a transferring stage between the two accelerated growth periods (infant and adolescent). Body dimensions are related to physical growth and development. The purpose of this study was to investigate physical growth in terms of anthropometry, muscle growth of the lower extremity, and functional development over early childhood.

**Methods:**

A cross-sectional study was carried out on 29 preschool children (PS: 3–5 years), 21 school children (SC: 6–8 years), and 22 adults (AD: 20–35 years). Lower extremity characteristics (segmental dimensions, muscle and adipose tissue thicknesses of the thigh and lower leg), and voluntary joint torque (knee and ankle) were measured. Correlations between parameters and group comparisons were performed.

**Results:**

All the parameters except for body mass index (BMI) and subcutaneous adipose tissue thickness were correlated with age for PS and SC combined (*r* = 0.479–0.920, *p* < 0.01). Relative thigh and shank lengths to body height were greatest in AD and smallest in PS (*p* < 0.05) but the relative foot dimensions were significantly larger in PS and SC than in AD (*p* < 0.05). Relative subcutaneous adipose tissue thickness was largest in PS and lowest in AD. Muscle thickness and the muscle volume measure (estimated from muscle thickness and limb length) were significantly larger in older age groups (*p* < 0.05). All groups showed comparable muscle thickness when normalized to limb length. Joint torque normalized to estimated muscle volume was greatest for AD, followed by SC and PS (*p* < 0.05).

**Conclusions:**

Relative lower extremity lengths increase with age, except for the foot dimensions. Muscle size increases with age in proportion to the limb length, while relative adiposity decreases. Torque-producing capacity is highly variable in children and rapidly develops toward adulthood. This cross-sectional study suggests that children are not a small scale version of adults, neither morphologically nor functionally.

## Introduction

Growth and functional development as a function of chronological age have often been examined from birth to adolescence [1–4]. Between those instances, the age from three to eight years is defined as early childhood, and constitutes a period with relatively slower changes in body dimensions compared to the two growth phases before and after it that typically show accelerated growth rate [2, 5]. However, some growth-related parameters, such as bone dimensions, do not systematically change with chronological age [6–8], and the patterns of change can be different for muscle size [9–11], subcutaneous adipose tissue [9, 12, 13], and muscle strength [14, 15]. Different structures demonstrate distinct patterns of the growth curves. The leg length becomes longer as compared to body height during the year of growth [16]. Muscle and adiposity develop in size with advancing age [9, 13]. A newborn is fatty and reaches the adiposity peak during infancy, then declines in the period of childhood [13]. In contrast, skeletal muscles continually grow from infancy to adulthood [9]. In early childhood, segmental growth, muscularity, and adiposity might change to a greater degree than that of the whole body dimensions and may even result in greater changes in strength development. However, this issue has not been studied to date.

Along with the body dimensional changes with age [17], there is substantial inter-individual variability in body size within a specific age range. This could confound the understanding of muscular growth and functional development [18]. However, the effect of body dimensions on muscular growth and development has been ignored or inconsistently accounted for [16, 19–22]. The maturation of muscle strength can occur, not due to an increase in the specific force of the muscle but to changes in muscle size, moment arm length, and neuromuscular function [23]. Normalization by body size has been applied to growth and development parameters including the segmental length relative to body height [16], muscle size to the related segmental length [19], and muscle strength to muscle size [20] or body mass [21]. Nevertheless, it remains unclear whether the differences of inhomogeneous growth and functional development among early childhood and adult persist after relevant variables are normalized to body size. The purpose of this study was to investigate characteristics of physical growth (body height, body mass, segmental dimension, muscle thickness, adiposity) and muscle strength, with and without body dimension normalization, in early childhood (preschool and school children) and adult. We hypothesized that there would be specific characteristics and patterns of lower extremity growth and development during early childhood when the body dimensional change is taken into consideration.

## Methods

### Study design and participants

The study employed a cross-sectional observational design that was conducted at Waseda University (Tokorozawa campus). A total of 50 healthy children voluntarily participated in the study and assigned to one of two groups: preschool children (PS: 3–5 years; 22 boys and 7 girls) or school children (SC: 6–8 years; 11 boys and 10 girls). In addition, 22 adults (AD: 20–35 years; 12 men and 10 women) voluntarily participated in this study as a reference. The inclusion criteria were both genders and the participants were excluded if they had any chronic disease or injury to the lower extremity or were on continuous medication.

The research was carried out in keeping with the Declaration of Helsinki for Human Subjects. This study was approved by the Human Research Ethics Committee of Waseda University (reference number: 2017–233). Prior to the examination, all participants (and their parents) were informed of the purpose and procedures of the study, and then informed consent was obtained.

### Procedures and Equipment

Our study has provided anthropometric data, muscle thickness, subcutaneous adipose tissue thickness (SAT), and lower extremity joint torque. Muscle size and adiposity measures as well as joint torque was normalized to segmental dimensions where applicable to remove the body-size dependence.

#### Anthropometry and morphology measurements

The anthropometric and morphology parameters in this study consisted of two categories: 1) general parameters: body height, body mass, and body mass index (BMI); and 2) segmental dimensions: the segmental dimensions of the lower extremity measured in a relaxed standing position. The measurement sites were located and marked, and the segmental lengths were measured including thigh length (the distance from the greater trochanter of the femur to the articular cleft between the femur and the epicondyle of tibia), shank length (the distance from the articular cleft between the femur and the epicondyle of tibia to lateral malleolus), and leg length (the distance of sum of thigh and shank lengths) [20]. Foot dimensions including foot length and foot height were determined by a three-dimension foot scanner (INFOOT, Japan). Body height was used to normalize the segmental dimensions.

#### Lower extremity muscle size and adiposity measurements

**Fig. 1:**
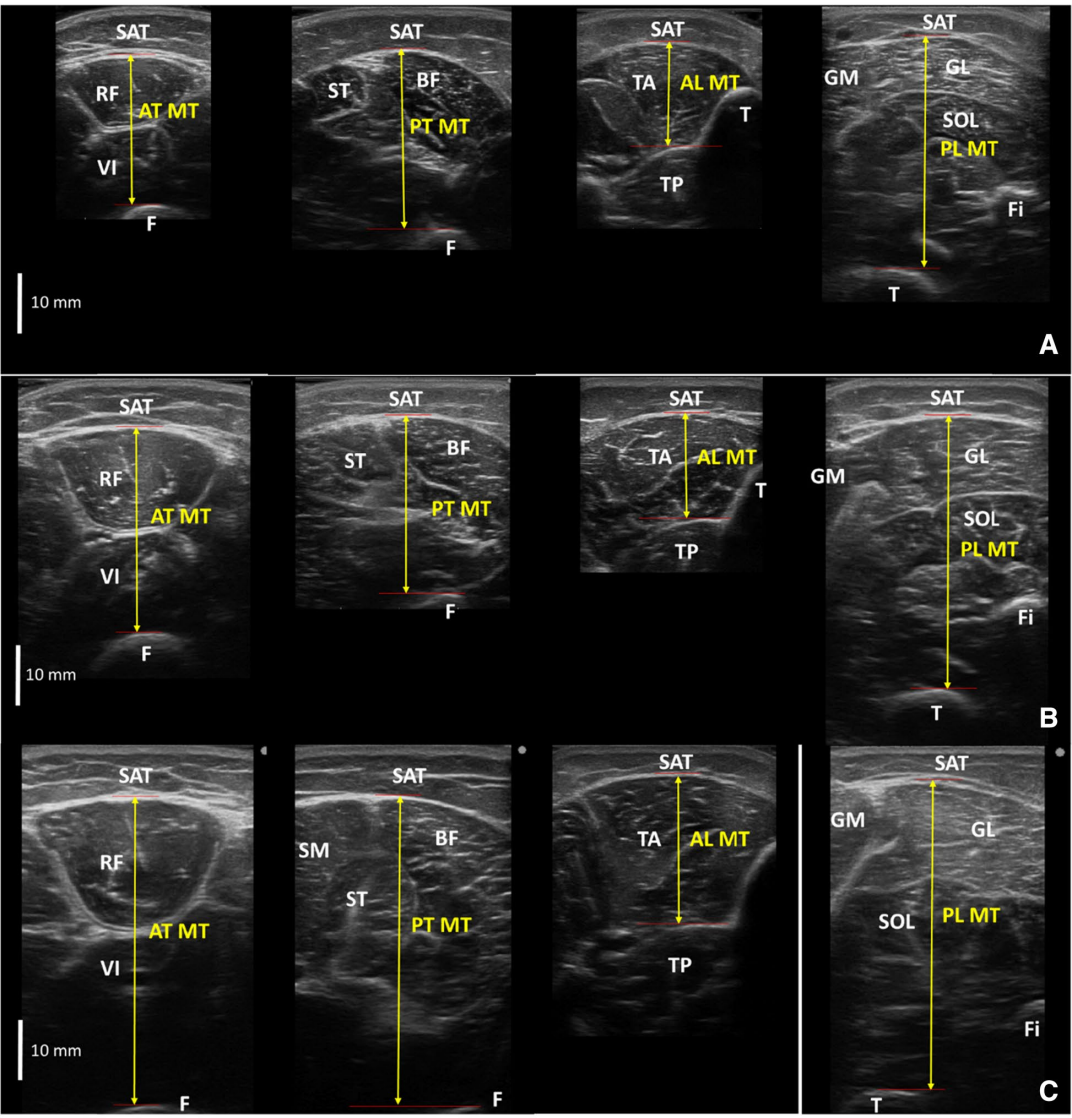
Ultrasonographic images of the lower extremity, **a** Preschool children, **b** School children, **c** Adults. AT: anterior thigh, PT: posterior thigh, AL: anterior lower leg, PL: posterior lower leg, MT: muscle thickness, SAT: subcutaneous adipose tissue thickness, RF: rectus femoris, VI: vastus intermedius, F: femur, ST: semitendinosus, BF: biceps femoris, TA: tibialis anterior, TP: tibialis posterior, GM: gastrocnemius medialis, GL: gastrocnemius lateralis, SOL: soleus, T: tibia, Fi: fibula

A transverse image of the muscle group located at the anterior thigh, posterior thigh, anterior lower leg, and posterior lower leg on the dominant limb was assessed using a B-mode ultrasonographic apparatus (Hitachi, Arietta Prologue, Japan) and a linear array probe with a scanning frequency of 7.5 MHz. The ultrasonography protocol was from the related study [20], and the images were obtained at 50% of thigh length and 30% of the proximal shank length for both anterior and posterior sites. All ultrasonographic measurements were performed in a relaxed standing position. Muscle thickness was defined as the distance between the adipose tissue-muscle interface to the muscle-bone interface. SAT was also measured at each site. Thigh and shank lengths were used to normalize the muscle thickness and SAT in the thigh and lower leg, respectively. Ultrasonographic images are presented in Fig. 1 as an example. The muscle thickness squared and multiplied by segmental length was used to estimate muscle volume in this study.

#### Muscle strength measurement

Maximum voluntary isometric joint torque was measured on the dominant leg in knee extension (KE), knee flexion (KF), ankle dorsiflexion (DF), and ankle plantar flexion (PF) using a dynamometer (Vine, Japan) and a specially designed myometer (Takei Scientific Instruments, Japan) for adults and children, respectively. For the knee measurements, each subject was positioned with the knee and hip joints at 90 degrees of flexion. In the ankle measurements, the knee was fully extended, and the hip at 90 degrees of flexion, and the ankle at neutral and 30 degrees of plantar flexion for PF and DF tests, respectively. For all measurements, the participants were appropriately stabilized with a non-elastic belt and two maximal efforts were performed for five seconds. In this study, the coefficient of variation was 3.8–10.4%, 3.7–8.5, and 2.9–3.6% for PS, SC, and AD, respectively. Verbal encouragement was given during the measurement. Estimated muscle volume (muscle thickness^2^*segmental length) was applied to normalize individual joint torque.

### Statistical analysis

The statistical software SPSS (Version 24.0, IBM, SPSS Inc., Chicago, USA) was conducted for statistical analysis of the data. Means and standard deviations (SD) were calculated for all parameters. The correlations among variables were determined using Pearson’s correlation coefficient or Spearman’s rank coefficient according to the variables’ distribution with the bootstrap procedure. Kruskal-Wallis One-way ANOVA was used to compare differences among three groups with Bonferroni correction to further analyze the significant results. The level of significance was set as a *p*-value <0.05.

## Results

Table 1 presents the correlation coefficient values of the physical growth and development with age and group comparisons. In early childhood, age was positively correlated with body height, body mass, segmental dimension, muscle thickness, and joint torque (*r* = 0.479–0.920, *p* < 0.01). However, BMI and SAT in the lower extremity were not correlated with age. Differences in absolute values of all parameters except BMI, foot height, anterior thigh muscle thickness, SAT, knee joint torque, and DF torque between PS and SC were statistically significant.

**Table 1:** Absolute growth and development parameters, correlation with age and comparison among three groups

Parameters	Children (*n* = 50)	Preschool children^a^ (*n* = 29)			School children^a^ (*n* = 21)			Adults^a^ (*n* = 22)		*p*
	*r*	Mean (SD)	Min–Max	*r*	Mean (SD)	Min–Max	*r*	Mean (SD)	Min–Max	
Age (year)		4.5 (0.9)	3.2–5.8		7.2 (0.9)	6.0–8.6		24.1 (3.4)	20.0–31.6	abc
General growth										
Body height (cm)	0.920**	104.1 (7.4)	89.0–116.6	0.822**	120.9 (7.1)	108.2–134.4	0.792**	166.2 (8.2)	152.1–178.5	abc
Body mass (kg)	0.849^a^**	16.6 (2.4)	12.4–21.0	0.654**	23.6 (4.6)	16.8–32.7	0.631**	60.0 (7.4)	48.6–76.7	abc
BMI (kg/m^2^)	0.225	15.3 (1.0)	13.6–17.6	- 0.303	16.0 (2.0)	13.3–21.0	0.256	21.7 (2.2)	17.2–26.9	bc
Segmental dimension										
Thigh length (cm)	0.902**	22.0 (2.3)	17.5–25.5	0.844**	26.7 (1.9)	23.0–31.5	0.677**	38.3 (2.2)	34.5–42.5	abc
Shank length (cm)	0.892**	22.1 (2.4)	17.5–26.0	0.841**	26.7 (1.9)	23.0–31.0	0.637**	37.8 (2.2)	34.0–42.0	abc
Foot length (cm)	0.844**	16.8 (1.0)	15.3–18.9	0.626**	19.1 (1.4)	16.3–22.9	0.755**	24.4 (1.6)	21.3–27.1	abc
Foot height (cm)	0.525**	4.5 (0.3)	3.5–5.1	0.133	4.8 (0.4)	4.1–5.9	0.588**	6.2 (0.5)	5.4–7.4	bc
Leg length: body height	0.637^a^**	0.42 (0.02)	0.37–0.45	0.801**	0.44 (0.02)	0.42–0.47	0.126	0.46 (0.01)	0.44–0.49	abc
Foot height: foot length	- 0.371**	0.26 (0.02)	0.22–0.30	- 0.333	0.25 (0.02)	0.22–0.29	0.141	0.23 (0.02)	0.19–0.29	bc
Muscular and adiposity										
AT MT (mm)	0.479**	28.0 (3.9)	18.9–34.5	0.157	31.1 (4.7)	22.5–41.5	0.499*	51.2 (3.6)	43.9–60.1	bc
PT MT (mm)	0.756**	32.5 (3.5)	24.0–37.5	0.526**	38.4 (4.2)	30.2–44.3	0.559*	57.9 (7.9)	42.9–71.7	abc
AL MT (mm)	0.748**	14.5 (1.2)	12.0–16.6	0.179	17.3 (2.0)	14.2–21.0	0.781**	28.1 (2.0)	25.0–33.8	abc
PL MT (mm)	0.794**	40.0 (3.7)	31.1–46.7	0.512*	44.9 (5.9)	34.2–55.5	0.683**	65.0 (7.9)	54.7–85.8	abc
AT SAT (mm)	0.199	7.6 (1.5)	4.7–10.9	- 0.218	8.7 (2.7)	5.3–13.5	0.115	8.8 (4.6)	3.0–17.5	ns
PT SAT (mm)	0.106	7.3 (1.9)	3.0–11.2	- 0.363	8.5 (3.2)	5.0–17.5	- 0.277	10.2 (5.5)	4.0–22.7	ns
AL SAT (mm)	- 0.145	4.1 (1.0)	2.6–6.5	- 0.242	4.1 (1.3)	2.0–7.7	- 0.454	3.5 (1.5)	1.7–6.5	ns
PL SAT (mm)	0.228	5.6 (1.1)	3.3–8.3	- 0.103	6.5 (1.7)	4.5–9.9	- 0.201	6.4 (2.8)	2.5–12.0	ns
AT MT^2^*Thigh length (cm^3^)	0.684**	174.0 (57.8)	62.6–303.5	0.384	268.3 (89.9)	133.7–465.0	0.558*	1007.7 (164.4)	782.1–1498.2	abc
PT MT^2^*Thigh length (cm^3^)	0.872**	235.1 (67.7)	100.8–358.6	0.741**	404.0 (102.8)	210.1–573.4	0.644**	1305.2 (350.4)	745.0–1926.0	abc
AL MT^2^*Shank length (cm^3^)	0.853^a^**	46.8 (11.6)	29.5–71.5	0.492*	82.2 (21.6)	47.2–117.0	0.798**	301.8 (50.3)	231.3–438.5	abc
PL MT^2^*Shank length (cm^3^)	0.861^a^**	306.8 (92.2)	169.5–566.2	0.738**	558.4 (172.8)	274.3–954.9	0.694**	1632.6 (457.4)	1029.5–2873.3	abc
Strength										
KE torque (Nm)	0.604^a^**	10.2 (6.3)	2.2–25.1	0.317	21.8 (13.1)	6.8–47.4	0.556*	184.2 (43.3)	121.3–255.1	bc
KF torque (Nm)	0.506^a^**	6.4 (4.6)	1.6–17.9	0.361	12.5 (7.6)	3.5–32.0	0.519*	74.5 (16.7)	40.8–101.3	bc
DF torque (Nm)	0.552^a^**	3.5 (2.3)	0.6–8.5	0.396	5.8 (3.4)	1.9–13.7	0.676**	31.3 (6.3)	19.6–40.4	bc
PF torque (Nm)	0.637^a^**	9.9 (5.7)	1.7–22.0	0.492*	20.7 (11.2)	6.1–37.9	0.495*	149.6 (39.0)	94.2–216.4	abc
KF: KE	- 0.041	0.65 (0.27)	0.30–1.50	0.155	0.61 (0.22)	0.29–1.17	- 0.025	0.42 (0.12)	0.30–0.79	bc
DF: PF	- 0.308^a^*	0.37 (0.12)	0.18–0.66	- 0.179	0.30 (0.12)	0.16–0.64	0.146	0.22 (0.06)	0.14–0.32	b

All parameters were analyzed by Pearson’s correlation coefficient except ^a^: Spearman’s rank coefficient; * = Significant correlation at *p* < 0.05, ** = Significant correlation at *p* < 0.01; Significant differences among three age groups are shown with a *p* < 0.05 (a, b: significant difference between preschool children to school children and adults, respectively, c: significant difference between school children and adults, ns: no significant difference)

*BMI* body mass index, *AT* anterior thigh, *PT* posterior thigh, *AL* anterior lower leg, *PL* posterior lower leg, *MT* muscle thickness, *SAT* subcutaneous adipose tissue thickness, *KE* knee extension, *KF* knee flexion, *DF* dorsiflexion, *PF* plantar flexion

**Fig. 2:**
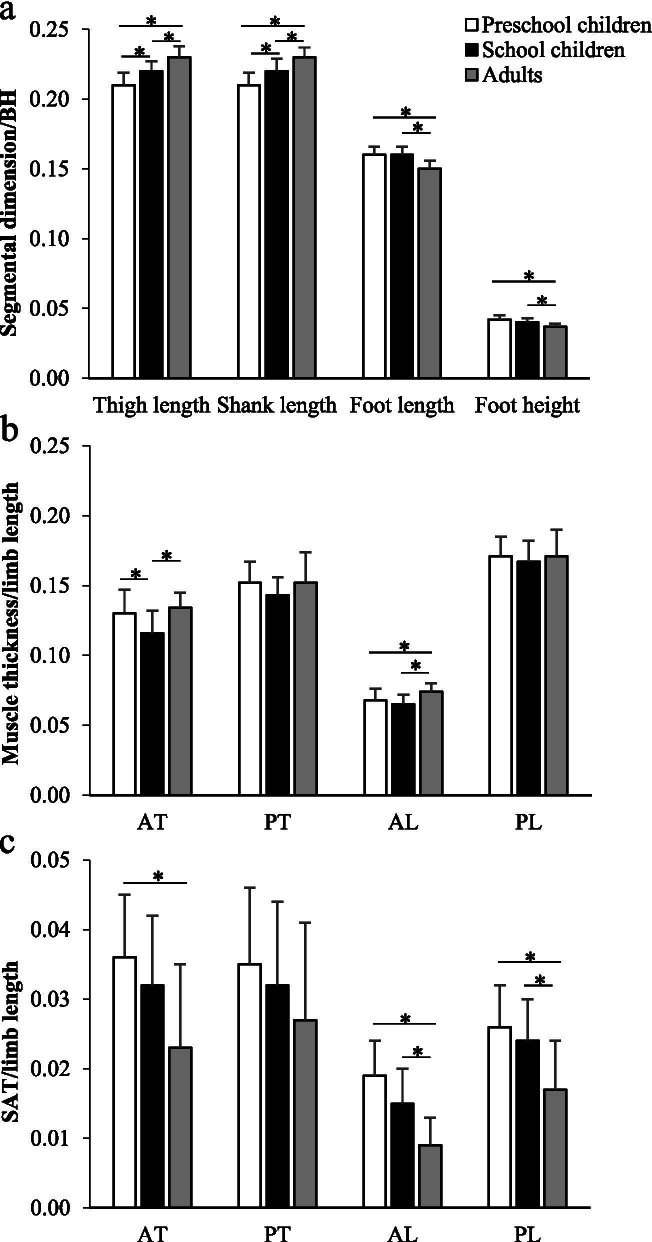
Comparisons of relative physical growth parameters, **a** segmental dimension (normalized to body height), **b** muscle thickness and **c** subcutaneous adipose tissue thickness (normalized to limb length). *: significant difference at *p* < 0.05, BH: body height, AT: anterior thigh, PT: posterior thigh, AL: anterior lower leg, PL: posterior lower leg, SAT: subcutaneous adipose tissue thickness

Figure 2 illustrates the comparisons of physical growths relative to body dimensions among the groups. Normalized thigh and shank lengths were greater in SC than in PS (*p* < 0.05). Foot length and foot height were significantly lower in AD when compared with PS and SC (*p* < 0.05). Lower extremity muscle thickness relative to segmental length showed a similar tendency among the three age groups. However, muscle growth trended greater in PS than in SC in the anterior thigh in particular (*p* < 0.05). For the relative SAT, the lower values were found in AD than in PS and SC (*p* < 0.05).

**Fig. 3:**
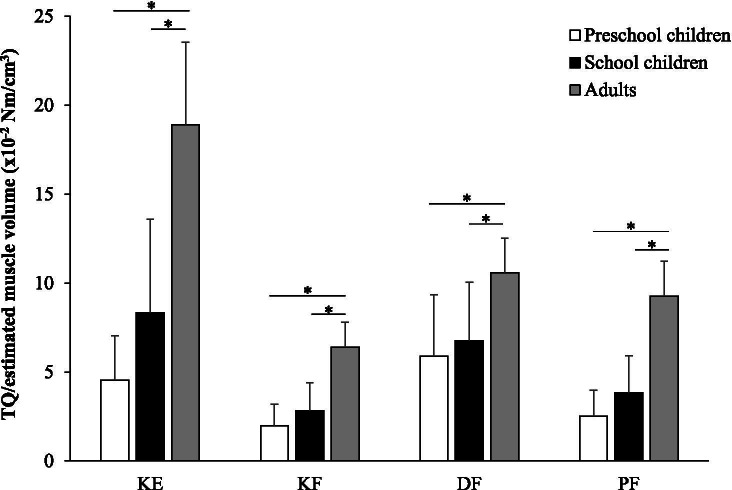
Comparison of joint torque/estimated muscle volume among three age groups. *: significant difference at *p* < 0.05, KE: knee extension; KF: knee flexion; DF: dorsiflexion; PF: plantar flexion, TQ: joint torque

After normalizing to estimated muscle volume, joint torque was significantly higher in AD than in PS and SC (*p* < 0.05) for all joints. However, there was no difference between PS and SC, in any of the specific joint torque (Fig. 3).

## Discussion

### General growth, segmental dimensions, muscle size, and adiposity

Body height and body mass were a function of age both in PS and SC. These results are in accordance with the WHO guideline for children [1]. Higher correlations among age and body size in the longitudinal dimension (body height and segmental lengths) were found in PS than in SC. This finding is in line with the fact that velocity of growth is higher from birth to five years of age, followed by a decline thereafter [2]. However, the lack of any association between BMI and age suggests that changes in body dimensions, musculature, and adiposity do not occur proportionally. Body height and body mass, but not BMI, could reflect the growth in early childhood.

Relative thigh and shank lengths were longer for older age groups. The thigh was of similar length to shank among the three groups. Gait characteristics develop with better posture and balance in older than in younger children [24] that may influence the total body length growth (lower extremity, trunk, and head). The proportion of leg length to total body length becomes greater with age until adulthood [16]. These findings suggest that changes in the relative segmental lengths occur over PS and SC.

A novel finding in our study is the pattern of foot growth in early childhood. Foot length and foot height were more strongly correlated with age in SC than in PS. The relative foot dimensions declined with age, in line with the change of foot length relative to body height [8]. Interestingly, our study found that the relative foot height was greater in PS and SC than in AD, while both PS and SC showed the similar values. The foot arch is developed with increasing age [25] which may explain the foot height development. However, a flat foot is a normal finding in early childhood periods [25, 26]. In addition, there was no association between increased body mass and foot arch in children [27]. Finally, the relative lower extremity muscle and adiposity were larger in PS than in SC and AD. Overall, these results suggest that the foot height may be mostly influenced by muscle and adiposity.

The absolute muscle thickness and estimated muscle volume increased with age. In contrast, the relative muscle growth was comparable over the three groups, except for the relative anterior thigh muscle thickness which was larger in PS than in SC. Skeletal muscle grows in multiple dimensions (mass, girth, and length) [4] as it adapts to various stimuli, including segmental skeletal length growth, physical activity, and exercise. Muscle growth is affected by chronological age and body dimension during early childhood, as previously seen from birth to puberty [3, 4, 10]. Muscle size develops similarly in magnitude to the respective skeletal segments, while the relative muscle growth in the transverse direction seems to develop similarly with the longitudinal segmental growth.

Regarding the adiposity changes, chronological age did not influence SAT in the lower extremity, as seen in children between 1–5 years of age [12]. Considering the absolute SAT, there were no systematic differences among the three age groups, at any measurement site. After adjusting body size to the related segmental length, adiposity drastically decreased with age, especially in the anterior thigh and lower leg, however, PS and SC were similar trends. Newborns have greater amount of adipose tissue which then declines with age, potentially due to changes in their nutrition and locomotor skills [13]. Overall, this study indicated that the relative adiposity declined with advancing age.

### Specific torque-generating capacity

An additional novel finding from this study is that the torque-producing capacity in early childhood rapidly increased toward adulthood, although normalized muscle thickness was similar between children (PS and SC) and AD, except for AT and AL where differences were found between SC and AD. This finding contradicts the notion that a muscle generates force in proportion to its size [28, 29]. It appears that muscle increases first in size and then in strength during early childhood. Our study also showed that the relative muscle size in the transverse direction may not fully account for the strength development in children in line with the previous studies [4, 30]. Factors that influence strength development include appropriate nutrition, growth hormone secretion, and nervous system development, such as motor unit recruitment and activation, and coordination of agonist and antagonist muscles [4, 23, 31–33]. These factors may explain the larger variability and the lack of significant subgroup differences in joint torque, except for PF, both in absolute and relative terms, in children in this study. Although the average absolute joint torque was sizably different between PS and SC. The proportion of gender in PS in this study may have affected the results, however, no gender differences have been found in strength until 10 to 12 years old [34, 35]. Daily physical activity and/or training can also affect strength development [36], although our participants were not routinely involved in any specific or vigorous exercise. Moreover, we found that KF:KE and DF:PF decreased with age. It is speculated that joint extension strength (both knee and ankle) develops with age more so than flexion strength, due to gross motor development [37].

Consistent with our hypothesis, our results supported that there were specific characteristics of lower extremity growth and strength in PS and SC. As the previous literature proposed, body size related differently on the outcome of performance and recommended the normalization method with respect to individual body dimension [18, 23, 38]. Considering the growth pattern with the body dimension in early childhood, the inhomogeneous structures including skeletal segment, muscle, and adiposity revealed different patterns that may impact muscle development. Muscular growth occurs in both longitudinal and transverse directions [4]. In PS the muscle growth in the transverse direction as well as segmental length may be the main effect on the joint torque. While the segmental length may influence the strength in SC that also relates to the longitudinal direction change.

This study presented some limitations that need to be considered. First, this was a cross-sectional observational study, and a longitudinal study is needed to further examine the pattern of children’s growth and development. Also, this study did not assess muscle activation, which may help explain the pattern of specific tension changes. Furthermore, indices of gross motor development, such as locomotor performance, were not treated in the present study but are worth investigating for a better understanding of physical growth and functional development in early childhood.

In conclusion, the specific growth patterns in early childhood demonstrate that the relative lower extremity lengths increase with age, while foot growth declines. Absolute muscle growth increases with age, however, the relative muscle thickness seems to remain unchanged among the three age groups. Strength capacity rapidly develops toward adulthood that is mainly impacted from muscle growth in transverse as well as in longitudinal directions for PS, and from muscle growth in longitudinal direction for SC, while it exhibits large variability in children. The results of the current study suggest that morphological development precedes functional development, and that children are not a small scale version of adults, neither morphologically nor functionally.

## Data Availability

The datasets generated and/or analyzed during the current study are available from the corresponding author on reasonable request.

## Abbreviations

PS: School children
SC: School children
AD: Adults
ANOVA: Analysis of variance
BMI: Body mass index
BH: Body height
AT: Anterior thigh
PT: Posterior thigh
AL: Anterior lower leg
PL: Posterior lower leg
MT: Muscle thickness
SAT: Subcutaneous adipose tissue thickness
RF: Rectus femoris
VI: Vastus intermedius
F: Femur
ST: Semitendinosus
BF: Biceps femoris
TA: Tibialis anterior
TP: Tibialis posterior
GM: Gastrocnemius medialis
GL: Gastrocnemius lateralis
SOL: Soleus
T: Tibia
Fi: Fibula
KE: Knee extension
KF: Knee flexion
DF: Dorsiflexion
PF: Plantar flexion
